# Age-Dependent Levels of Protein Kinase Cs in Brain: Reduction of Endogenous Mechanisms of Neuroprotection

**DOI:** 10.3390/ijms20143544

**Published:** 2019-07-19

**Authors:** Donatella Pastore, Francesca Pacifici, Kunjan R. Dave, Raffaele Palmirotta, Alfonso Bellia, Guido Pasquantonio, Fiorella Guadagni, Giulia Donadel, Nicola Di Daniele, Pasquale Abete, Davide Lauro, Tatjana Rundek, Miguel A. Perez-Pinzon, David Della-Morte

**Affiliations:** 1 Department of Systems Medicine, University of Rome Tor Vergata, 00133 Rome, Italy; 2 Department of Neurology, The Evelyn McKnight Brain Institute, Miller School of Medicine, University of Miami, Miami, FL 33136, USA; 3 Department of Biomedical Sciences and Human Oncology, University of Bari “Aldo Moro”, 70124 Bari, Italy; 4 Policlinico Tor Vergata Foundation, University Hospital, 00133 Rome, Italy; 5 Department of Clinical Sciences and Translational Medicine, University of Rome Tor Vergata, 00133 Rome, Italy; 6 Department of Human Sciences and Quality of Life Promotion, San Raffaele Roma Open University, 00166 Rome, Italy; 7 Department of Translational Medical Sciences, University of Naples, Federico II, 80138 Naples, Italy

**Keywords:** protein kinase c, aging, ischemic preconditioning, brain, neurodegenerative diseases, modulators, pharmacogenetics, Alzheimer’s Diseases, cerebrovascular disease

## Abstract

Neurodegenerative diseases are among the leading causes of mortality and disability worldwide. However, current therapeutic approaches have failed to reach significant results in their prevention and cure. Protein Kinase Cs (PKCs) are kinases involved in the pathophysiology of neurodegenerative diseases, such as Alzheimer’s Disease (AD) and cerebral ischemia. Specifically ε, δ, and γPKC are associated with the endogenous mechanism of protection referred to as ischemic preconditioning (IPC). Existing modulators of PKCs, in particular of εPKC, such as ψεReceptor for Activated C-Kinase (ψεRACK) and Resveratrol, have been proposed as a potential therapeutic strategy for cerebrovascular and cognitive diseases. PKCs change in expression during aging, which likely suggests their association with IPC-induced reduction against ischemia and increase of neuronal loss occurring in senescent brain. This review describes the link between PKCs and cerebrovascular and cognitive disorders, and proposes PKCs modulators as innovative candidates for their treatment. We report original data showing εPKC reduction in levels and activity in the hippocampus of old compared to young rats and a reduction in the levels of δPKC and γPKC in old hippocampus, without a change in their activity. These data, integrated with other findings discussed in this review, demonstrate that PKCs modulators may have potential to restore age-related reduction of endogenous mechanisms of protection against neurodegeneration.

## 1. Introduction: Protein Kinases C

Protein Kinase Cs (PKCs) was discovered more than 30 years ago as the receptor of a natural cancer-promoting agent, the phorbol ester [1], and was firstly found as a single proteolitically-activated kinase in rat brain [2]. PKCs family belongs to the superfamily of AGC (protein kinase A/protein kinase G/protein kinase C-family) Ser/Thr (Serine/Threonine) kinases and is composed of 10 characterized members [2,3,4]. By considering their domain structure and required cofactors for their activation, PKCs can be divided into three main groups: 1. conventional (cPKC: PKCα, β1, β2, and γ), which are regulated by diacylglycerol (DAG), phospholipids and calcium (Ca2+); 2. novel (nPKC: PKCδ, ε, η, θ) that need only DAG to be regulated; and 3. atypical (aPKC: PKCζ, ι), which are independent from both DAG and Ca2+ [1,2]. All PKCs show a regulatory region in the N-terminus and a catalytic domain at the C-terminus; both of which are composed of a conserved domain (C1 and C2 located in the N-terminal, C3 and C4 in the C-terminal region), containing the functional domains and poorly conserved (or variable) regions (V1–V5) [2,5]. In particular, the C1 domain is highly conserved in all PKCs members. It represents the DAG binding site and the phosphatidylserine (PS) domain, responsible for the PKC–membrane interaction and subsequent PKCs activation. The C2 region is the Ca2+ binding site and is present only in the cPKC [5]. The C3 region allows ATP (Adenosine Triphosphate) binding while the C4 domain is considered the catalytic core [2,5]. They are both highly conserved in all members of the family [5]. Moreover, all PKC enzymes contain a pseudosubstrate domain, which maintains them in an inactive state [6]. 

Once PKCs are translocated, they are bound to the membrane in an inactive conformation [3]. The complete activation of the enzymes requires three different phosphorylation processes. The first is mediated by the Phosphoinositide-dependent kinase-1 (PDK-1), which phosphorylates PKCs in the activation loop (Thr500 for the cPKC; Thr566 for the nPKC; Thr410 for the aPKC) [7], allowing the subsequent phosphorylation in the C-terminus domain which in turn leads to the completely active enzyme [3]. The second process is represented by an autophosphorylation in the turn motif in order to maintain the catalytic competence of the enzymes (Thr641 in cPKC; Thr710 for nPKCδ, and θ and Ser710 for the nPKCε and η; Thr560 for the aPKC) [7]. A dephosphorylation process on this site induced PKC inactivation and promotes its degradation by the ubiquitin-proteasome system [3]. To avoid the inactivation of PKC’s enzymes, the heat shock protein Hsp70 binds to the turn motif site of PKCs, stabilizing the protein and allowing a novel phosphorylation and a re-activation of PKCs [8]. Finally, the third process is a phosphorylation involving the hydrophobic motif (Ser660 for the cPKCα, β1, β2 and Thr660 for cPKCγ; Ser729 for the nPKC; Glu for the aPKC) [7], which ultimately leads to the mature and active enzyme [3]. After these phosphorylation events, PKCs are located in the cytoplasm and could be fully activated by second messengers. In particular, for cPKCs, Ca2+ binds to theC2 domain, pre-targeting the enzymes to the cell membrane [9]. The subsequent association of the C1 domain with DAG allowed cPKCs to release the pseudosubstrate domain in order to bind with the substrate and initiated the downstream signaling pathway [3]. The nPKCs lack the C2 domain, but their C1 domain has an increased affinity for DAG leading to a direct and stronger association with membrane compared to the C1 domain of cPKCs [3]. Since the aPKCs are independent from both Ca2+ and DAG, they are regulated and activated only by phosphorylation processes [10]. As previously mentioned, PKCs activation follows their translocation from cytosol to plasma or other cellular membranes. This process is mediated by the interaction of PKCs with scaffolding proteins called receptors for activated C-kinase (RACKs), which properly localizes the enzyme nearby the target substrates [11]. Other scaffolding proteins are the phosphoserine/threonine binding protein 14-3-3 and the A-kinase anchoring proteins (AKAPs) [12]. The 14-3-3 proteins bind and activate εPKC; furthermore, they have also an inhibitory effect [12]. In lens epithelial cells, in fact, 14-3-3 proteins bind to γPKC leading to a reduction in its activation either by promoting the inactive conformation or by sequestering the enzyme away from its substrates [12]. Regarding AKAPs scaffolding proteins, they may have an inhibitory effect, such as AKAP12 that binds and inactivates δPKC and α, or may contribute to the maturation and stabilization of PKCs increasing their activation [12]. 

**Figure 1:**
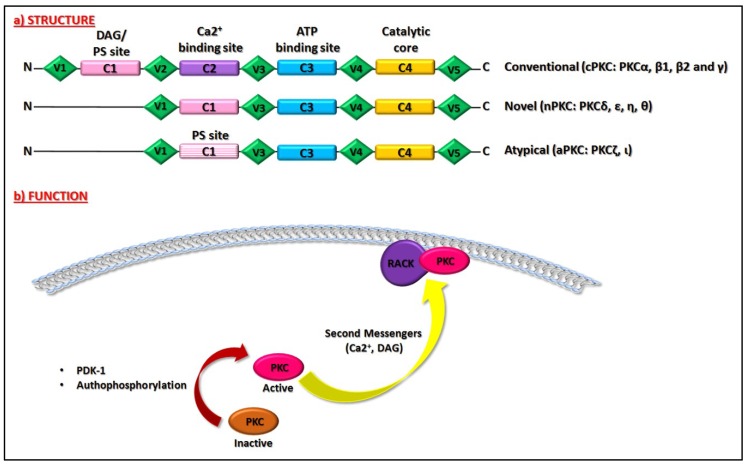
Structure and Function of PKCs. The domain structure of PKCs family proteins (**a**). Schematic representation of different domains in Conventional, Novel and Atypical isoforms of PKCs. PKCs present in the N-terminus (N) a regulatory region and a catalytic domain at the C-terminus (C), composed by a conserved domain (C1 and C2 located in the N-terminal, C3 and C4 in the C-terminal region), which contains the functional domains and variable regions (V1–V5). C1 domain is highly conserved in all PKCs members. It represents the DAG binding site and the phosphatidylserine (PS) domain, responsible for the PKC–membrane interaction and subsequent PKCs activation. The C2 region is the Ca2+ binding site and is present only in the cPKC. The C3 region allows ATP binding, and the C4 domain is a catalytic core. Description of PKCs function (**b**). The complete activation of PKCs requires mainly phosphorylative processes mediated by the Phosphoinositide-dependent kinase-1 (PDK-1), which phosphorylates PKCs, and an autophosphorylation in order to maintain the catalytic competence of the enzymes. After these phosphorylative events, PKCs could be activated by second messengers such as Ca2+ and DAG. PKCs activation follow their translocation from cytosol to plasma or other cellular membranes. This process is mediated by the interaction of PKCs with scaffolding proteins called receptors for activated C-kinase (RACKs), which properly localizes the enzyme nearby the targeted substrates. The arc in blue and white represents the cellular plasma membrane. The red curved arrow denotes the activation of PKC following phosphorylation processes. The yellow curved arrow showed PKC translocation to plasma membrane induced by second messengers.

PKCs activation could be reversed by reducing Ca2+ content or by activating the DAG kinase, which phosphorylates DAG, leading to phosphatidic acid formation, which is unable to activate PKCs [3,13]. Moreover, once activated, PKCs are more susceptible to the action of protease and phosphatase, which in turn, reduces PKCs activity [3,14]. Among phosphatases, the recently discovered family of PH domain Leucine-rich repeat Protein Phosphatase (PHLPP) dephosphorylates PKCs on the hydrophobic site, reducing its stability, leading to protein degradation [15]. Like other proteins, PKCs are also susceptible to ubiquitin proteasome degradation; the activated phosphorylated form of PKCs seems to be necessary for their degradation [16]. Furthermore, the phosphorylation on proline (P), glutamate (E), serine (S), and threonine (T) residues, also known as the PEST site, which is expressed in all PKCs and is common to other proteins, is responsible for targeting PKCs to proteasome degradation [17]. All these processes deeply regulate the catalytic activity of PKCs enzymes (Figure 1).

## 2. Protein Kinase Cs in Brain and Neurological Diseases

Neuronal tissue showed increased PKCs activity and expression. The activation of these kinases has a positive effect in the control of several brain functions, such as ion channel regulation, receptors modulation, promoting neurotransmitters release, synaptic potentiation/depression, and neuronal survival—all are involved in neurological disorders [18]. Moreover, PKCs play a central role in regulating a wide variety of physiological neuronal processes, from cell growth to learning and memory [18,19]. Especially cPKCs have been implicated in controlling brain functions and physiological processes through the phosphorylation of a large variety of substrates in neurons. Therefore, they control many different signaling cascades, such as actin cytoskeleton, microtubule dynamics, GSK-3β (Glycogen synthase kinase 3), AMPA (α-amino-3-hydroxy-5-methyl-4-isoxazolepropionic acid) receptor, and NMDA (N-methyl-D-aspartate) receptor pathways, which are all involved in synaptic plasticity [20].

Loss in PKCs expression and function have been indicated as one among the first markers of neuronal death [21]. Therefore, impairment in PKCs is surely implicated in neurological age-related disorders, such as cerebral ischemia, brain injury, and cognitive impairment [4,18]. 

In the latest decades, it was proven that different brain areas express PKC isoforms differently [22]. The cerebellum expresses the largest amount of cPKCs including all of its four isoforms. However, despite their high prevalence in the brain, the physiological and pathological roles of cPKCs in cerebellum, especially in Purkinje cells, have not been fully understood [23]. The hippocampus presents other expression patterns of PKCs [18]. A study investigated the cytosolic and particulate fractions of cortex, striatum and hippocampus obtained from young and middle-aged rats showing no significant changes between young and middle-aged animal brains, therefore suggesting the changes in their expression may begin since middle-age and continue in aged brain [24]. Similar findings were also recently reported [25]. However, a sex-specific difference on PKCs brain distribution with an increase in expression in male brain, which deserves further investigation, has been suggested [25]. Moreover, it was also suggested that this age-related change in their activity and expression may explain, at least in part, an increase of chronic diseases susceptibility in the elderly [26,27]. To better understand the role of PKCs in the brain and their expression throughout a lifespan, it is important to explore their behavior in different neurological diseases. 

### 2.1. PKCs, Role in Ischemic Preconditioning and in Brain Ischemia 

Brain ischemia and reperfusion is characterized by a cerebral region that is distal to an artery occlusion. This region undergoes tissue necrosis with cell death ensuing within a few minutes of ischemia onset [28]. Multiple cellular mechanisms are rapidly activated in response to ischemia-reperfusion (I/R) injury. Among those, inflammation, oxidative stress with increase in reactive oxygen species (ROS) production, reduction in cellular ATP level, and mitochondrial impairment, are the most important [29]. Different PKCs isoforms are implicated in all of these processes [30]. Mainly three PKCs isoforms play a pivotal role in neurons in ischemic injury and tolerance, εPKC, δPKC, and γPKC [30]. 

Activated εPKC exerts a protective role against cerebral ischemic/reperfusion damage. It was proposed to be among the main mediators of the Ischemic Preconditioning (IPC) [4,31]. IPC refers to an endogenous mechanism of protection whereby brief sub-lethal periods of ischemia are able to reduce the deleterious effects of a subsequent, longer duration of ischemic episodes [31,32]. This mechanism of protection has been demonstrated to occur in several organs, such as heart, brain, liver, and kidney [33]. When triggered by endogenous or exogenous stimuli or IPC, εPKC translocates from cellular cytosol to cellular particulate fractions, such as mitochondrial and nuclear membranes [30]. There, when activated, by binding its receptor RACK1, it regulates many pathways implicated in IPC-induced neuroprotection, including phosphorylation of mitochondria K^+^_ATP_ channels, increasing synaptosomal mitochondrial respiration and activation of the extracellular signal-regulated kinase (ERK) pathway [30]. In the brain, εPKC interacts with several neuronal receptors and neuromediators including N-methyl-d-aspartate (NMDA), gamma-aminobutyric acid (GABA) synapses, BDNF (Brain-derived neurotrophic factor), SIRT1 (Sirtuin1), and SIRT5 [34,35]. The neuroprotective properties of εPKC-mediated IPC have been reported by using εPKC agonists (ψε receptors for activated C kinase-RACK, εPKC activator peptide; εPKC85–92:CHDAPIGYD) and εPKC inhibitor (εV1-2). Intravenous injection of ψεRACK (εPKC agonist) 30 min before the induction of ischemia was protective in the hippocampal CA1 neurons from lethal cerebral ischemic damage induced by a two-vessels occlusion model in rats. Measurements of Cerebral Blood Flow (CBF) before, during and after cerebral ischemia revealed a significant reduction in the reperfusion phase in rats pretreated with ψεRACK compared to the control group. These results suggested that the activation of εPKC reduced ischemic/reperfusion damage by a significant decrease in blood flow during reperfusion after ischemia [32]. Similar protective evidences for εPKC were reported by using an Oxygen Glucose Deprivation (OGD) model, (in vitro ischemia) highlighting the role of NMDA receptors in cerebral ischemic tolerance [36]. 

Conversely, δPKC and γPKC play a noxious role when activated during cerebral ischemic/reperfusion damage [31]. Mochly-Rosen along with Perez-Pinzon’s collaborators demonstrated that the selective inhibitor of δPKC, δV1-1, significantly reduced cellular injury in a rat hippocampal model after OGD and during the first 3 h of reperfusion [37]. Perez-Pinzon’s group, moreover, clearly showed that the inhibition of δPKC decreased infarct size in an in-vivo rat stroke model of transient middle cerebral artery occlusion (MCAO), particularly by decreasing apoptosis, increasing levels of phospho-Akt, and inhibiting BAD (Bcl-2-associated death) protein translocation, indicating inhibition of proapoptotic signaling after I/R injury [37]. Similar findings for δPKC were also reported in rats by using a model of global ischemia (asphyxial cardiac arrest—ACA) [38]. Moreover, rats treated with δV1-1 (pre- and post-ischemia) exhibited improved perfusion after 24 h and less hippocampal CA1 neuronal death 7 days after ACA, suggesting a cerebral blood flow modulation mechanism linked to δPKC during I/R processes [39]. 

Similar to δPKC, inhibition of γPKC by Go6983 has been demonstrated to decrease OGD-induced increment in LDH (Lactate dehydrogenase) leakage and decrease the cell survival rate in hippocampal slices [40]. However, controversial results reported that γPKC knockout mice (PKCγ−/−) significantly increased the infarct volume and neuronal cell loss in the peri-infarct region and enhanced the neurological deficits, the impaired coordination, and the reduced muscle strength of mice following 1 h MCAO/1–7 day reperfusion [41]. This protection has been suggested to be mediated by G-protein-coupled estrogen receptors [42]. Therefore, the role of γPKC in the brain I/R mechanism is not still fully clarified. 

### 2.2. PKCs, Alzheimer’s Diseases and Cognitive Disorders

Since PKCs-related pathways have been involved in the control of memory and learning processes, their role in cognitive disorders were investigated [43,44,45,46]. These cognitive disorders include Alzheimer’s Disease (AD), Parkinson’s Disease (PD), Vascular Dementia, and Huntington Disease (HD). AD is the most common cause of dementia and is characterized by a chronic loss in memory and neurological functions consequently by a reduction in cholinergic neurons associated with a plaque deposit of extracellular β amyloid (Aβ) and intracellular neurofibrillary tangles [47]. The prevalence of AD, like other types of cognitive disorders, increases exponentially along with age [47]. In the brain of patients with AD, the levels of PKCs are significantly reduced and PKCs signaling is impaired in terms of activity and translocation to the cellular membrane [48,49]. Therefore, a direct effect of PKCs in AD, as an etiopathogenic cause or as consequence of neurological damage, is highly suggestive [44]. The most significant results have been reported that the αPKC, γPKC and εPKC isoforms are altered since they are the most related to synaptic transmission and memory formation [50]. These PKCs regulate neurotransmission and synaptic plasticity by phosphorylating transporters, ion channels, and G protein-coupled receptors. PKCs phosphorylate and regulate the dopamine transporter, α-amino-3-hydroxy-5-methyl-5-isoxazolepropionic acid (AMPA)-type glutamate receptors (AMPARs), NMDA-type glutamate receptors (NMDARs), γ-aminobutyric acid (GABA) receptors, μ-opioid receptor, and metabotropic glutamate receptor 5 (mGluR5) receptors [51,52,53]. Interestingly, a recent study conducted in a SH-SY5Y cell line, in brain cortical region samples from patients with AD, and in transgenic APPswe/PS1dE9 mice which develop AD, demonstrated an increase in δPKC activity and expression that regulates β-site APP-cleaving enzyme 1 (BACE1) expression, thereby enhancing Aβ production. δPKC inhibition resulted in a protection against Aβ neuropathology and in a significant rescue against cognitive deficits, indicating that δPKC inhibition may be a viable treatment strategy in AD.

The role of γPKC has been also shown in PD, where a suppression of this kinase through dominant-negative mutant or small interfering RNA, effectively blocked apoptotic cell death in an in-vitro model of PD [54]. Similarly, elevated levels of δPKC have been associated with an increase in intranuclear huntingtin aggregates in a transgenic model of HD [55]. These studies suggest that different levels of PKCs are associated with different susceptibilities to develop neurological diseases. Moreover, a link with neurodegeneration has been further proposed for RACK1 and RACK2, which bind specifically to PKCs and serves as adaptor proteins for several other signaling enzymes that regulate the PKCs-mediated effects [56]. RACK2 is the εPKC-specific RACK and is a coated-vesicle protein involved in transcellular pathways regulation [56]. A decrease in RACK1 distribution in the membrane fraction of cortical neurons was linked with an increase in Aβ oligomers [57], suggesting that the PKC-RACK signal transduction complex may be pivotal in the pathophysiology in AD.

## 3. Modulators of PKCs: Focus on εPKC

Based on the previous evidence, the modulation of the PKCs isoforms may be an interesting therapeutic approach in stroke and cognitive decline. Numerous molecules such as proteins, lipids, and second messengers that interact with different PKCs domains, particularly with εPKC, modulate their function and activity [11]. A study conducted on ventricular myocytes isolated from guinea pig hearts, investigated the effects of εPKC and δPKC on the modulation of the sarcolemmal adenosine triphosphate-sensitive potassium (sarcK_ATP_) channel by anesthetic isoflurane [58]. Isoflurane alone was unable to open the sarcK_ATP_ channel; however, pre-treatment with a specific εPKC activator, PP106, induced εPKC translocation in both mitochondria and sarcolemma, resulting in the opening of the sarcK_ATP_ channel [58]. Differently, the δPKC activator PP114 was significantly less effective in priming the sarcK_ATP_ channel after anesthetic-induced preconditioning, since it is translocated only in the mitochondria [58]. Specific compounds, such as indolactam and benzolactam, have been proposed as nPKCs activators due to their capacity to bind to the C1 domains of PKCs in a selective manner [4,59]. After hemorrhagic shock, the activation of both αPKC and εPKC via adenosine A1 receptor (A1 receptor) in rats, promoted cardio- and neuroprotection through IPC [60]. 

Other interesting molecules able to modulate PKCs activity are bryostatins, in particular bryostatin 1 family member [61]. The bryostatins are a family of complex macrolactone natural products and are powerful PKCs agonists [61]. Bryostatin 1 has been shown to reverse synaptic loss and facilitate synaptic maturation by activating εPKC in animal models of AD, Fragile X, stroke, and other neurological disorders [62]. Pre-clinical mouse studies showed effective bryostatin 1 activation of εPKC and increased levels of BDNF (Brain-derived neurotrophic factor) and PSD-95 (postsynaptic density protein 95). Moreover, similarly to bryostatin 1, εPKC activator DCPLA (dicyclopropanated linoleic acid) methyl ester prevented and/or reversed synaptic loss in an animal model of aging [62]. Bryostatin 1 activates PKCs, and particularly αPKC and ε, by binding to the C1 and C2 domains. In particular, it produces a time-dependent biphasic effect on εPKC, characterized by an initial activation, followed by εPKC membrane translocation. 

Several natural molecules, such as Resveratrol, a polyphenol present in strongly pigmented vegetables and fruits and red wine, modulate PKCs activity [63]. Resveratrol has different biochemical and physiological functions, including estrogenic, antiplatelet, anti-inflammatory, anti-cancer, and antioxidant [63]. Resveratrol prevents diabetes, obesity, and metabolic syndrome and protects against atherosclerosis and cardiovascular disease (CVD) [64]. Resveratrol was found to increase the activity of the anti-aging molecule SIRT1, a NAD (Nicotinamide adenine dinucleotide) +-dependent histone deacetylase [65]. In an in-vivo study, we demonstrated that Resveratrol induced neuroprotection by mimicking the effect of IPC [66]. In particular, εPKC played a key role in regulating the mitochondrial NAD+/NADH (Nicotinamide adenine dinucleotide dehydrogenase) ratio, following IPC and Resveratrol administration. These findings suggest that εPKC is pivotal in inducing the Resveratrol-mediated neuroprotective effects in rat brain [66]. 

There are also several ATP-competitive small molecule inhibitors, such as Balanol [67], Riluzole [68], Staurosporin, H7 and Chelerythrine that block all PKCs isoforms although they are too toxic for clinical use [69,70]. Indolcarbazole and bisindoylmaleimide have shown to have selectivity to specific PKCs isoforms [70]. Other inhibitors that compete at the DAG/phorbol ester or the PS binding site, may be more specific, including Calphostin C that binds to the C1 domain, mimicking DAG-association [64]. Moreover, peptides that inhibit PKCs activation, such as the myristoylated peptide myr-ΨPKC, and peptides that disrupt protein/protein interactions between the PKCs regulatory domain and RACK, have been developed [69,71]. The interaction of PKCs and its receptor RACKs is isoform-specific and is largely mediated by the C2 region. Peptide fragments of this region have been developed as modulators of PKCs activity [72]. These short peptides induce activation and translocation of the corresponding PKCs isoforms by mimicking the action of RACKs and are, therefore, termed ‘pseudo RACKs’ (ΨRACK) [73,74]. Disruption of the interaction between ΨεRACK and RACK-binding site is a critical rate-limiting step in the translocation and activation of εPKC [75]. 

Other compounds that counteract the effects of PKCs include activators of β-adrenoceptors and antioxidants, such as seleno compounds, vitamin E, and curcumin [4]. In 1996, Mochly-Rosen [76] identified an εPKC-selective antagonist, called εV1-2 peptide, that disrupts PKCs binding to its receptor, RACK2, and inhibits εPKCtranslocation and function in cardiac myocytes. εV1-2 peptide abolished hypoxic preconditioning and phorbol ester-mediated cardiac protection mediated by εPKC [77]. Therefore, considering the number of pathologies in which PKCs are involved, and how their functions can be crucial in pathophysiological processes, specific molecules that regulate their activity, may be considered as highly innovative therapeutic strategies.

## 4. Pharmacogenetics of PKC Modulators: Focus on εPKC

Clearly, a therapeutic approach with PKCs modulators is innovative and quite experimental. In the context of this new approach, pharmacogenetics studies are needed to understand how genetic polymorphisms, or genetic variants, in genes coding for functional proteins involved in pharmacodynamics, influence PKC-modulating therapeutic effects [78]. To date, there are only a few studies on the association between genetic variants and therapeutic responses using PKCs target drugs, and almost all of them are focused on εPKC. Scientific data are limited to preclinical studies using cell lines or animal models. However, data provided by these experimental studies are preliminary but promising to identify molecular targets for pharmacogenetics application involving PKCs modulation that can be applied in precision medicine.

An in-vitro study suggested an important role of sulfotransferases (SULTs) isozymes genetic variants on Resveratrol disposition [79]. SULTs act as catalysts of conjugation for various endogenous substrates, such as hormones, neurotransmitters, proteins, carbohydrates, and xenobiotics, as well as therapeutic drugs, carcinogens and polyphenols present in the diet [80]. Among the various families of sulfotransferase enzymes, the SULT1A isoenzyme catalyzes the sulfonation of catecholamines and phenolic derivatives [81]. To date, there are no clinical studies of association between SULT1A polymorphisms and the use of resveratrol, probably also due to the fact that these polymorphisms present a great ethnic variation between Asian, Caucasian and African populations [82].

The uridine diphosphate glucuronosyltransferases (UGT) is a superfamily of detoxifying enzymes involved in the glucuronidation of resveratrol, and the main recognized isoforms that catalyzes this metabolic reaction are UGT1A1 and 1A6, and to a lesser extent, 1A9 [83,84]. A research examined the association between the resveratrol glucuronidation and the presence of three non-synonymous cSNPs (coding Single Nucleotide Polymorphisms) in the first exon of the UGT1A6 gene (c.19T > G p.Ser7Ala-rs6759892; c.541A > G p.Thr181Ala-rs2070959 and c.552A > C p.Arg184Ser-rs1105879) and the polymorphic TA6/7 repeat in the UGT1A1 promoter (rs34983651), showing an association between variants on UGT and resveratrol metabolism [84]. 

Recently, the relation between the effects of resveratrol and the enzyme manganese superoxide dismutase (SOD2) polymorphism c.47C > T (Ala16Val-rs4880) [85] has been investigated. The presence of the T allele (Valine) is responsible for the presence of an instable mRNA with reduction of the enzyme transport into the mitochondrial matrix and consequent poor antioxidant function [86]. In human peripheral blood mononuclear cells (PBMC) cultures treated with resveratrol, Ala16 variant was related also to a decrease in cell proliferation and production of inflammatory cytokines, thus proving that the Val16 variant may play a role in proliferation and inflammatory cytokine secretion [85].

Amongst the modulators of PKCs activity, Isoflurane has been reported to exert a cardioprotective effect mediated by εPKC-inducing pre-conditioning [87]. The mechanism by which Isoflurane-activated PKCs has been reported to be ERK1/2 (Extracellular signal-regulated kinases1/2) phosphorylation- and CaMKII (Ca^2+^/calmodulin-dependent protein kinase II) activation-dependent [88]. In human liver microsomes, it has been shown that Isoflurane is metabolized for a small proportion by CYP2E1 (Cytochrome P450 2E1) to trifluoroacetic acid [89]. Variant CYP2E1* 5 (−1293G > C; −1053C > T), which is known to increase the transcription of the gene, may influence the metabolism of the molecule. However, no studies have investigated the induction of CYP2E1 based on its genotype [89].

A study performed on 80 ICR/CD-1 (Institute for Cancer Research/Caesarean Derived-1) mice divided into isoflurane-sensitive (S group) and resistant (R group) strains showed a close association between a SNP (Single Nucleotide Polymorphisms) at the nucleotide position 462 (C/G) of the β1 GABAA (gamma-aminobutyric acid) receptor subunit and the state of resistance to isoflurane [90]. The finding that C (cytosine) at nucleotide 462 had a greater frequency in the resistance group (36 mice from the resistant and only 5 mice from the sensitive strain) suggested that the polymorphism may alter the sensitivity of animals to isoflurane by modulation of its binding capability [90].

More recently, a large pharmacogenetic study was performed on different strains of Drosophila Melanogaster by evaluating the effect of isoflurane using a Serial Anesthesia Array apparatus. The results indicated that mutant strain ND2360114 was much more sensitive to anesthetic compared to other strains, leading to the conclusion that this mutation confers resistance to isoflurane [91]. The Drosophila Melanogaster ND23 nuclear gene, a homologue of the NDUFS8 gene in humans, is highly conserved among eukaryotes and prokaryotes and encodes a core subunit of Complex I of the mitochondrial electron transport chain [92]. Therefore, it is presumed that in humans, a reduced activity of the electron transport chain determined by gene alterations may proportionally affect the sensitivity to isoflurane as already hypothesized in previous studies [93].

## 5. Age-Related Differences of PKCs in Brain 

We previously discussed how a deficit of PKCs levels and activation was associated with acute and chronic neurological disorders [4]. Aging is the main risk factor for vascular disease and cognitive decline [94]. Aging increases the brain’s susceptibility to many pathological processes. For example, brain damage following stroke is greater in elderly individuals [95] and leads to mortality rates three times higher in aged individuals compared to younger individuals [96]. In addition, the aging brain is more susceptible to neurodegenerative diseases such as AD [97]. How aging is involved in these changes is not fully understood. 

### 5.1. Aging and Ischemic Preconditioning 

Aging leads to loss of viability, an increase in vulnerability, and a progressive decrease in the endogenous mechanisms of defense. IPC is one of the most important endogenous protective mechanism against ischemia, but it decreases with aging [98]. An in-vivo study using young and aged rats (4 and 24 months) subjected to IPC (3-min ischemia) followed by 10-min ischemia, through the assessments of histology and the immunoreactivity of N-methyl-D-aspartic acid receptor 1 and caspase-3 active peptide in the hippocampal CA1 region performed 8 days after full ischemia, clearly demonstrated that the degree of cerebral protection against ischemia was reduced in the aged and preconditioned rats compared with the young rat [99]. The clinical equivalent of cerebral IPC is the transient ischemic attack (TIA), a brief ischemic event occurring before a prolonged ischemic period in the same vascular territory leading to ischemic stroke [100]. In 203 patients aged 65 years or older with diagnosis of acute ischemic stroke, we reported no significant differences in the admission or discharge National Institutes of Health Stroke Status and modified Rankin scores between patients who had TIA within 72 h of stroke onset and those without TIA, suggesting that cerebral IPC is lost in the elderly [101]. 

Mechanisms to explain the age-related reduction of cerebral IPC may be multiple and include the physiological reduction in neurons, mitochondrial dysfunction, increase in oxidative stress, and alterations in hormonal profiles [98]. PKCs and their isoforms have been linked to IPC pathways of protection. A difference in activation/translocation and levels of ε, δ and γ PKCs, and RACK1 and RACK2 in the brain across ages may at least in part explain the age-related reduction of IPC and the higher susceptibility of aged brain to cognitive disorders. 

### 5.2. PKCs and Aging

Few studies have investigated PKCs levels and their activity in aged brain. The main assumption includes a decrease in function of all PKCs isoforms during aging [26]. This deterioration may be related to epigenetic modification [102], or to a decrease in the translocation from soluble to particulate cellular fractions along with a parallel reduction in RACK1 and RACK2 [103]. A comprehensive review of cerebral PKCs isoforms and aging has been conducted [49]. In the hippocampus, both βPKC and ζPKC were significantly reduced in aged rodents’ brains [104,105], while δPKC did not change the levels [106]. In the hippocampus, both αPKC and εPKC were reduced [107]. These alterations in PKCs levels have been more associated with a loss in memory and cognitive decline than with a loss of the IPC mechanism of protection against stroke during aging [49]. Moreover, a clear evaluation of the most important PKCs isoforms involved in cerebral ischemia and neurodegeneration across ages has not yet been performed.

### 5.3. Hippocampal PKCs Isoforms and RACKs Levels Across Age

Based on the previous literature gap, in this review we sought to report results on the levels of ε, δ and γ PKCs in the soluble and particulate/membrane compartments in rat hippocampus at three different age time points. We chose the hippocampus since (1) it is the region of the brain most vulnerable to ischemia [108] and (2) it plays an important role in AD [109]. We also determined levels of RACK1 and RACK2, as upon activation, PKCs binds to the respective RACKs in order to phosphorylate their target substrate proteins [110]. Young (4 month-old, Y, *n* = 4), middle-aged (12 month-old, M, *n* = 4), and old (24 month-old, O, *n* = 4) male Fisher344 rats were used in these experiments. These animal ages would reflect a human age of 18, 30/40, and >60 years old, respectively [111]. All procedures involving rats were approved by the University of Miami of Miami Animal Care and Use Committee on 07/01/2007 (Identification Number: 0725314B). Rats were anesthetized with 3% halothane and 70% nitrous oxide (in balanced oxygen) by inhalation and then sacrificed. The brains of the rats were removed and the hippocampi were isolated from each brain. At the time of Western blot analysis, the hippocampus was washed once with cold PBS (Phosphate Buffered Saline). Ten percent homogenate was prepared in homogenizing buffer (4 mmol/L ATP, 100 mmol/L KCl, 10 mmol/L imidazole, 2 mmol/L EGTA, 1 mmol/L MgCl2, 20% glycerol, 0.05% Triton X-100, 17 μg/mL PMSF, 20 μg/mL soybean trypsin inhibitor, 25 μg/mL leupeptin, 25 μg/mL aprotinin) using an all-glass homogenizer. The homogenate was then centrifuged at 4 °C at 1000 g for 10 mins. The supernatant is the soluble/cytosolic fraction and was carefully removed and recentrifuged at 16,000× *g* for 15 min to get rid of any contaminating pellet material. The initial pellet was resuspended in 250 μL of cell lysis buffer containing 1% Triton X-100 and was extracted on ice for 60 min. Samples were centrifuged at 16,000× *g* for 15 min. The supernatant was the particulate fraction. The soluble/cytosolic fraction was analyzed for protein contents using the Bio-Rad protein assay kit (Hercules, CA, USA)), based on the method of Bradford [112]. Protein was transferred to an Immobilon-P membrane and incubated with rabbit polyclonal antisera to δPKC (1:500); εPKC (1:500); γPKC (1:800), or monoclonal antibodies anti-RACK1 antibody (1:250) and anti-RACK2 antibody (1:250) and for β-actin (monoclonal anti-β–actin, 1:4000), to ensure equal protein loading. Following incubation with secondary antibodies, immunoreactivity was detected with an enhanced chemiluminescence (ECL) Western blotting detection kit. A one-way analysis of variance (ANOVA) followed by a multiple comparison procedure (Bonferroni’s test) was used to analyze differences among groups. Statistical analyses were carried out with Systat 7.0 software (Available online: www.systatsoftware.com). Results were considered statistically significant if *p* < 0.05.

**Figure 2:**
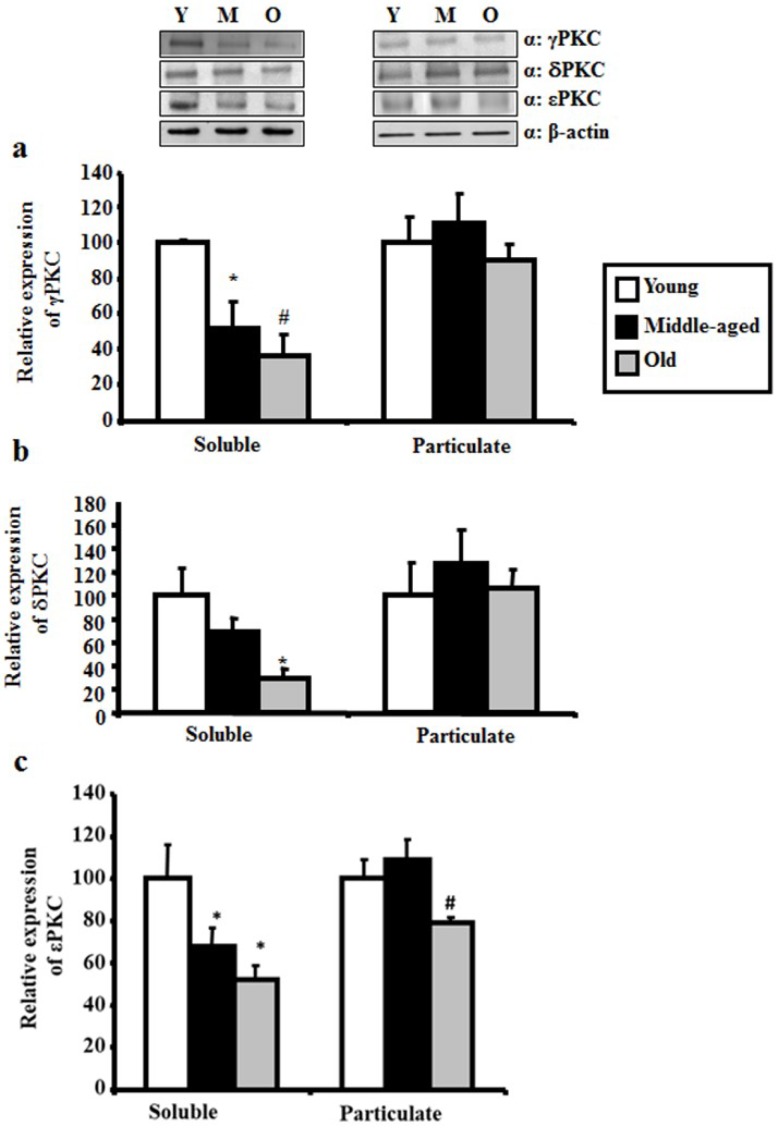
γPKC, δPKC and εPKC levels in hippocampus of young (Y), middle-aged (M), and aged rats (O). Western Blot analysis of γPKC, δPKC and εPKC levels in the soluble and particulate fractions of young, middle-aged, and old rats hippocampus. γPKC levels in the soluble fraction were significantly lower in old and middle-aged rats when compared to young rats, while no significant differences were present in particulate fraction (**a**). δPKC levels differ significantly between young and old rats in the soluble fraction while no significant differences were present in the particulate fraction (**b**). εPKC levels were significantly lower in the soluble fraction of old and middle-aged rat hippocampus compared to levels in young hippocampus. Also, εPKC levels were lower in the particulate fraction of old rat hippocampus compared to the young and middle-age groups (**c**). * *p* < 0.05 compared to the young group for γPKC, # *p* < 0.02 compared to the young group for γPKC, * *p* < 0.02 compared with the young group for δPKC and * *p* < 0.05 versus the rat young group. # *p* < 0.05 versus the rat young and middle-aged groups for εPKC (*n* = 4 for each group).

In soluble fractions of middle-aged and aged rat (old) hippocampi, γPKC levels were about 49% (51.48 ± 16.03, *p* < 0.05) and 64% (36.54 ± 12.82, *p* < 0.02) lower than the levels found in young rats, respectively (Figure 2a). The δPKC levels in soluble fractions of aged rat hippocampi were 70% (29.99 ± 8.14, *p* < 0.02) lower than in young rat hippocampi (Figure 2b). Finally, the εPKC levels in the soluble fractions of middle-aged and aged rat hippocampi were about 31% (67.89 ± 6.56, *p* < 0.05) and 48% (51.9 ± 6.98, *p* < 0.05) lower compared to young rat hippocampi, respectively (Figure 2c). In the particulate fraction, where activated PKCs translocate and bind, no significant differences were observed in γPKC and in δPKC expression among the three age groups (Figure 2a,b). However, εPKC levels were 21% and 30% lower in the particulate fraction of aged hippocampi (78.92 ± 2.85, *p* < 0.05) compared to the young (100 ± 8.68, *p* < 0.05) and middle-aged (108.85 ± 9.54, *p* < 0.05) groups respectively (Figure 2c).

**Figure 3:**
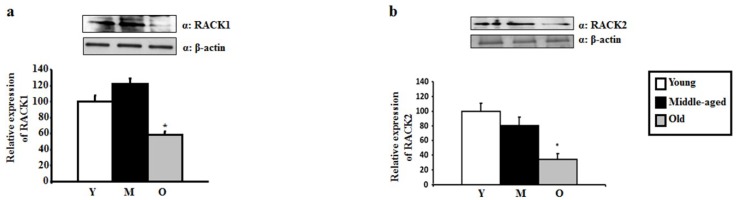
RACK1 and RACK2 levels in the hippocampus of young, middle-aged, and aged rats. Western Blot analysis of RACK1 (**a**) and RACK2 (**b**) expression in young (Y), middle aged (M) and old (O) rats hippocampus. RACK1 and RACK2 levels differed significantly between the aged rat group and the other two age groups. No significant differences in RACK1 and RACK2 levels were found between young and middle-aged groups.* *p* < 0.02 compared to the middle-aged group, and * *p* < 0.05 compared to the young group for RACK1. * *p* < 0.02 compared to the young group; and * *p* < 0.05 compared to the middle-aged group for RACK2 (*n* = 4 for each group).

**Figure 4:**
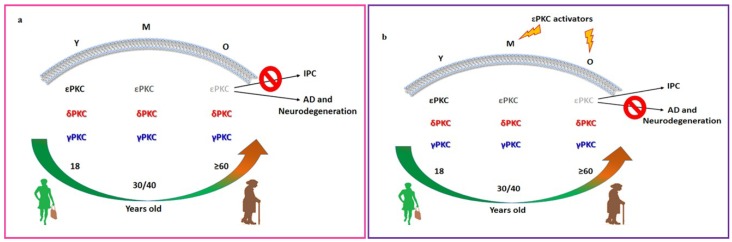
Illustrative representation of ε, δ and γ PKCs, and RACKs levels in particulate/membrane fractions of rat hippocampus at three different ages, young (Y), middle age (M) and old (O). A different level of ε, δ and γ PKCs in the brain across age may explain the age-related reduction of IPC protection and higher susceptibility of aged brain for cognitive disorders and neurodegeneration. These animal ages reflect a human age of 18, 30/40, and >60 years old, respectively. In the particulate fraction, where activated PKCs translocate, no significance differences were observed in γPKC (blue) and in δPKC (red) expression across the three age groups. However, εPKC (black) levels were lower in the particulate fraction of aged hippocampi compared to the young and middle-aged groups (as represented by the different color intensity, grey). This alteration may explain, at least in part, the greater incidence of AD and damage after cerebral ischemia in elderly individuals (**a**). The presence of εPKC activators has been demonstrated to restore IPC protection mechanisms in aged brain and to reduce susceptibility to AD and other neurodegenerative diseases where PKCs are involved (**b**). The red circle with a slash in it represents an inhibitory effect.

Since activation of PKCs leads to their translocation to the particulate fraction, where they bind with their respective RACKs, we next measured RACK1 and RACK2 levels. RACK1 (58.53 ± 7.2, *p* < 0.05) and RACK2 (34.14 ± 8.2, *p* < 0.02) levels in the aging hippocampus were 41% and 66% lower than RACK1 and RACK2 levels in the young hippocampus, respectively (Figure 3a,b). RACK1 (121.95 ± 3.7, *p* < 0.02) and RACK2 (80.48 ± 11.5, *p* < 0.05) levels were also respectively 63% and 43% lower in the aging hippocampus compared to the middle-aged hippocampus (Figure 3a,b). RACK1 and RACK2 levels were not significantly different between the young and middle-age groups (Figure 3a,b), (Figure 4).

### 5.4. Conclusions and Future Perspective

In this review, we reported a significant reduction in εPKC levels in both the soluble and particulate fractions in the hippocampus. Here, we also found a significant reduction in levels of δPKC and γPKC in the old hippocampus in the soluble but not in the particulate fraction, where δPKC and γPKC are active, and a significant reduction in RACK1 and RACK2 levels in the old hippocampus. However, we acknowledge the limitation that we did not determine the PKCs expression in the total sample of our animal subgroups, since we extrapolated these data from other studies [18,113]. All these proteins are involved in the pathophysiology of brain ischemia, AD, and IPC [4]; therefore, accordingly with previous findings, we demonstrated that aging produces changes that make the hippocampus more susceptible to potential cellular damage in response to stressors. The age-related reduction in IPC may be linked to the lower levels of εPKC in the aged hippocampus (Figure 4). Further study needs to be performed to validate this hypothesis. Moreover, lower levels of εPKC in the particulate fraction of the aged hippocampus might partly explain the increase in Aβ accumulation in the aged brain and the greater incidence of AD in elderly individuals (Figure 4). Lower RACKs levels thus result in impaired phosphorylation of target proteins. Our results are consistent with the previous results that showed a dramatic loss of RACK1 in the pellet fraction of the old rabbit hippocampus [114] and with findings reporting a reduction in a brain cortex RACK1 levels in old rats [106,113]. RACK2 also decreased in the aged rat heart [115]. A decrease in RACK2, demonstrated here, coupled with a decrease in the εPKC levels, can enhance the effects of PKCs impairment on the aged hippocampus. 

All these findings are of particular importance, since the presence of PKC modulators, especially εPKC activators, such as Ψε-receptors for activated C kinase and Resveratrol, has been demonstrated to mimic IPC and therefore should be tested as a therapeutic strategy to restore IPC mechanisms in aged brain. A similar approach may be used for AD and other neurodegenerative diseases where PKCs are involved. However, it is intuitive that further studies are imperative to better understanding the role of PKCs in aging and their impact on neurodegenerative and vascular disorders.
